# Cerebral Cortical Changes in Craniosynostosis: A Systematic Review

**DOI:** 10.7759/cureus.74575

**Published:** 2024-11-27

**Authors:** Michail Palaios, Christos Rapanos, Ioannis Kapsokavadis, Kyriaki Katouni, Dimitrios Filippou

**Affiliations:** 1 Department of Anatomy, National and Kapodistrian University of Athens School of Medicine, Athens, GRC; 2 Department of Biomedical Sciences, Research and Education Institute in Biomedical Sciences, Pireaus, GRC

**Keywords:** cerebral cortical changes, cerebral cortical thinning, genetic disease, neural connectivity, non-syndromic craniosynostosis, syndromic craniosynostosis

## Abstract

This study aims to review the existing literature on cerebral cortical changes in craniosynostosis during the months of August and September 2023. It focuses on alterations occurring in cases of both syndromic and non-syndromic forms of the disease. In particular, variations in volume, size, and structure (e.g., connectivity) of the cortex are studied.

For the present review, a systematic search of the PubMed database was performed using the terms "cerebral cortex"[tw] OR "Cerebral Cortex"[Mesh] AND "Craniosynostoses"[Mesh]. The initial search retrieved 50 articles, which were studied in their entirety. After applying the selection and exclusion criteria, 34 articles were excluded, and finally, 16 qualified and were included in this study. The Anatomical Quality Assessment (AQUA) tool was preferred for the quality assessment of the included publications.

Part of the articles used refers to the syndromic form of the disease and discusses temporal lobe and frontal cortex abnormalities, thinning and disproportionate increase in cerebral cortical surface area, and simplified gyroscopic pattern. The remaining articles, referring to non-syndromic craniosynostosis, are focused on neuronal connectivity, grey matter volume, and Sylvian fissure volume. In the existing literature, two theories have been proposed to describe the relationship between craniosynostosis and cortical changes. The deformation theory states that skull deformities result in brain architecture malformations, and the deformity theory supports that brain abnormalities pre-exist and lead to premature fusion of the cranial sutures.

The existing data are not sufficient to resolve the above dilemma. Regarding the therapeutic approach, it differs depending on the type of craniosynostosis. Surgery remains the most common method, while innovative treatments are also emerging, including the application of regenerative medicine.

## Introduction and background

Craniosynostosis is a developmental disorder associated with premature fusion of the cranial sutures [1,2]. It occurs in one out of 2,000-2,500 births and can be divided into syndromic, resulting from inherited genetic mutations, and non-syndromic, a disease with subtle genetic etiology [2,3]. The affected sutures can be sagittal, metopic, lamboid, and coronal, with sagittal craniosynostosis being the most common type [4]. Mutations in the fibroblast growth factor receptor (FGFR) and TWIST1 genes [1] or chromosomal abnormalities [2] cause the syndromic variants of the disease. As a result, different craniosynostotic syndromes (Apert, Crouzon-Pfeiffer, Muenke, and Saethre-Chotzen syndromes) occur [1,2]. On the other hand, non-syndromic craniosynostosis is non-heritable, is seen roughly in one per 1000 live births, and is described in 80% of all cases of the disease [3,5]. The developmental complications vary, depending on the type of craniosynostosis. Typical examples include motor and psychological disorders, organ development malformations, limb dysplasia, mental delay, facial abnormalities, and respiratory failure, which differ among the syndromes of craniosynostosis [1,6,7,8,9]. Visual disabilities and deafness are also possible problems [2,8,9]. Behavioral and emotional disorders, deficits in reading, language, and memory abilities, neurological dysfunctions, and developmental delays can be connected with non-syndromic types of craniosynostosis [4,10,11].

It is worth mentioning that surgery is indicated as the most common way of treating the disease. In this context, it is remarkable that many young patients with complex syndromic craniosynostosis undergo the frontofacial monobloc advancement surgical method [1,12].

This study aims to review the existing literature available from August to September 2023 on cortical changes in craniosynostosis, and presents these malformations in both syndromic and non-syndromic forms of the disease. The study specifically highlights the maldevelopments in cortical volume, size, and structure (such as neural connectivity) caused by craniosynostosis, elucidating the connection between craniosynostosis and cerebral cortical deformities.

## Review

Methods

The study was conducted during the months of August and September 2023. For its completion, we searched during the months mentioned before for scientific articles in the PubMed and ClinicalTrials.gov databases. The following search: "cerebral cortex"[tw] OR "Cerebral Cortex"[Mesh] AND "Craniosynostoses"[Mesh] generated 50 articles, all of which were studied. All of the authors contributed to the study selection and assessment of the review's quality. Palaios M., Rapanos C., and Kapsokavadis I. participated in data extraction.

The exclusion criteria were text not written in the English language; title not relevant to the subject; study not focused on human beings; not referred to craniosynostosis; no abstract available in PubMed or not yet recruiting/unknown status/suspended/withdrawn in ClinicalTrials.gov; abstract not relevant to craniosynostosis. The inclusion criteria were full text or abstract available on PubMed or recruiting/completed/terminated in ClinicalTrials.gov and contained case reports of human patients with cerebral cortex malformations in syndromic and non-syndromic craniosynostosis.

**Figure 1:**
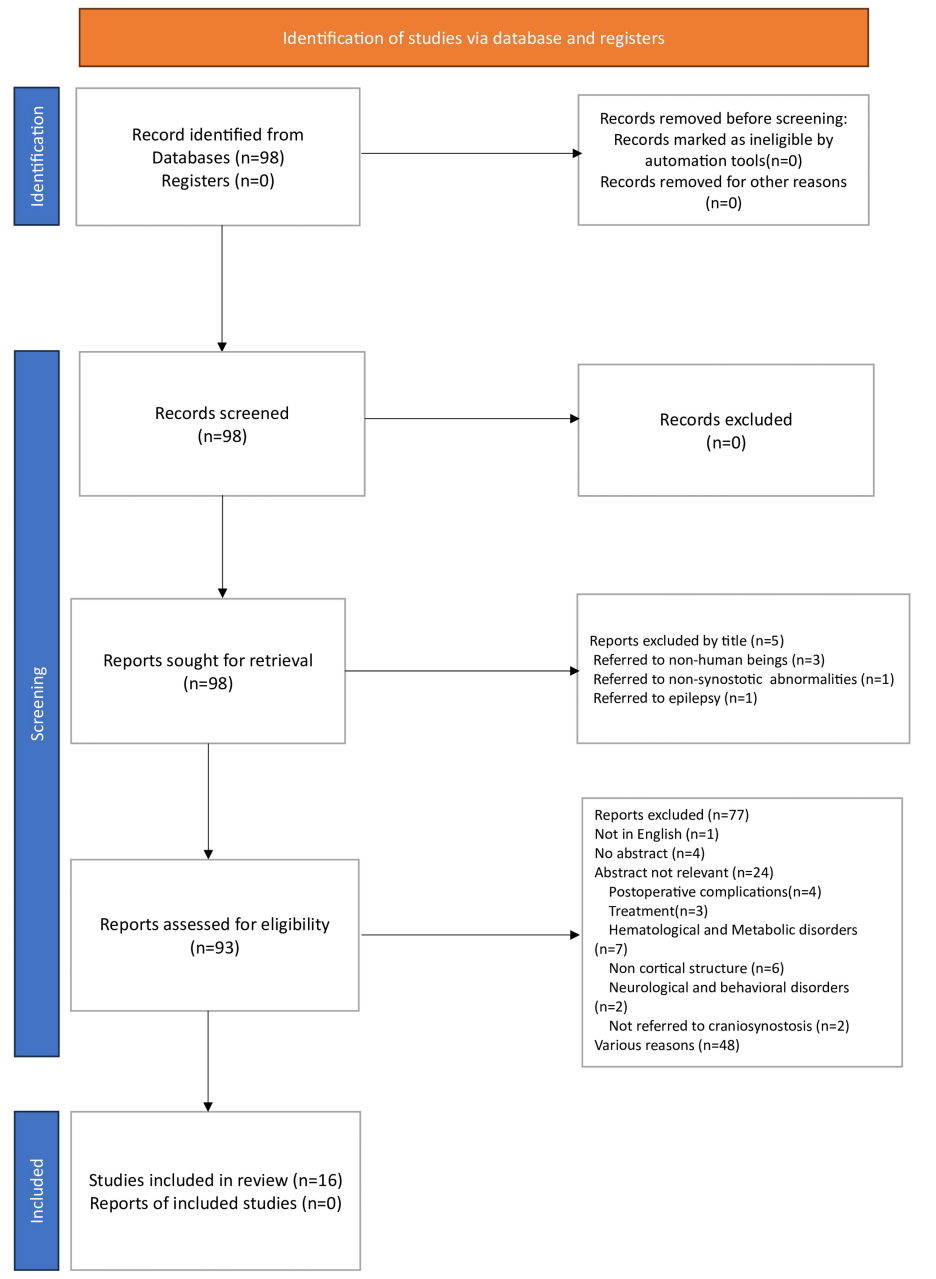
PRISMA flowchart PRISMA: Preferred Reporting Items for Systematic Reviews and Meta-Analyses

Having taken into consideration the criteria mentioned above, 50 publications were retrieved from PubMed and 48 from ClinicalTrials.gov; 82 publications were excluded for various reasons, while 16 were enrolled. A Preferred Reporting Items for Systematic Reviews and Meta-Analyses (PRISMA) flowchart, which is shown below, analyzes the processes of identification and screening (Figure 1). The present systematic review adheres to the PRISMA guidelines. The Anatomical Quality Assessment (AQUA) tool was employed to assess the quality of the included publications (Henry, 2017) [13]. The AQUA tool is designed to evaluate the risk of bias (RoB) in anatomical studies across five domains: objectives and subject characterization; study design; characterization; descriptive anatomy; and reporting of results. In each domain, questions are answered with ‘yes’ (Y), ‘no’ (N), or ‘unclear’ (U). In case a positive answer (Y) is accredited to every question within a certain domain, the related RoB is considered low (Table 1). Overall, each included study was assessed as “low RoB” in case <2 domains were considered as low RoB [13]. In detail, the quality assessment of the included studies is presented in Appendix 1.

**Table 1:** Risk of bias (RoB) assessment of the included studies using the AQUA tool estimating five domains Domain A (objectives and subject characterization); Domain B (study design); Domain C (methodology characterization); Domain D (descriptive anatomy); and Domain E (reporting of results); Y: yes, U: unclear, N: no; L: low RoB, H: high RoB; RoB: risk of bias; AQUA: Anatomical Quality Assessment

Publication	Domain A	Domain B	Domain C	Domain D	Domain E	Overall RoB
Wilson AT et al. [1]	Y	Y	Y	Y	Y	L
Stark Z et al. [6]	Y	Y	Y	Y	Y	L
Quintas-Neves M et al. [7]	Y	Y	Y	Y	Y	L
Tokumaru AM et al. [8]	Y	Y	Y	Y	Y	L
Grosso et al. [14]	Y	Υ	Υ	Υ	Υ	L
Abdel-Salam GM et al. [15]	Y	Y	Y	Y	Y	L
Okubo Y et al. [9]	Y	U	Y	Y	Y	L
Wider TM et al. [16]	Y	U	Y	Y	Y	L
Cobb AR et al. [12]	Y	Y	Y	Y	Y	L
Wilson AT et al. [2]	Y	Y	Y	Y	Y	L
Barge-Schaapveld DQ et al. [17]	Y	Y	Y	Y	Y	L
Park KE et al. [10]	Y	Y	Y	U	Y	L
Cabrejo R et al. [4]	Υ	Υ	Υ	U	Y	L
Beckett JS et al. [3]	Y	Y	Y	U	Y	L
Aldridge K et al. [11]	Y	Y	Y	Y	Y	L
Sawin PD et al. [5]	Y	Y	Y	Y	Y	L

Results

In total, sixteen of the initially 98 retrieved articles are included in this overview, all of which were focused on the consequences of craniosynostosis in the cerebral cortex. Eleven of the articles refer to the syndromic form of the disease, and five of them refer to the non-syndromic craniosynostosis (Table 2).

**Table 2:** Presentation of each article according to the topic referred

Craniosynostosis type	Consequence in the cerebral cortex	Articles
Syndromic	Temporal lobe abnormalities	6
	Frontal cortex deformities	2
	Thinning of the cerebral cortex	1
	Simplified gyroscopic pattern	1
	Disproportionate increase in the surface area of the cerebral cortex	1
Non-syndromic	Cortical neural connectivity	3
	Gray matter volume	1
	Sylvian’s fissure’s volume	1

Discussion

This review constitutes an overall attempt to summarize the existing literature on the changes in the cerebral cortex during craniosynostosis, following its distinction between syndromic and non-syndromic. By that means, information regarding both forms of the disease was collected in order to present their effect on the cerebral cortex in one paper.

Syndromic Craniosynostosis

Craniosynostosis is a congenital disorder characterized by premature fusion of the sutures of the skull that leads to the deformation of the shape of the skull, which is related directly to the involved sutures [1,2,4]. As a matter of fact, any pattern of cranial growth restriction poses a risk to the developing brain [1,4]. In syndromic cases of the disease, multiple sutures are often involved due to mutations in genes such as fibroblast growth factor receptor (FGFR) genes and the TWIST gene, which are vital for cortical and mesodermal development, respectively [1]. The incorrect expression of these genes might result in the development of various syndromes related to syndromic craniosynostosis, such as Apert, Crouzon-Pfeiffer, Muenke, and Saethre-Chotzen syndromes [1,2].

Due to the premature fusion of the neurocranial sutures seen in syndromic craniosynostosis, the underlying cerebral cortex is often affected in ways analyzed below (Table 3).

**Table 3:** Overview of each paper focused on syndromic and non-syndromic cases of craniosynostosis FGFR: fibroblast growth factor receptor

Νο	Title	Author	Year	Conclusion
1	Cerebral cortex maldevelopment in syndromic craniosynostosis	Wilson AT et al. [1]	2022	Patients with FGFR gene mutations, present less surface in regards to the volume in parietal and occipital lobes.
2	Apert syndrome: temporal lobe abnormalities on fetal brain imaging	Stark Z et al. [6]	2015	Individuals with Apert syndrome, due to mutations in the FGFR2 gene, present temporal lobe abnormalities.
3	Fetal brain MRI in Apert syndrome: early in vivo detection of temporal lobe malformation	Quintas-Neves M et al. [7]	2018	Brain MRI of fetus with Apert syndrome shows abnormalities in the lower and temporal lobe region.
4	Skull base and calvarial deformities: association with intracranial changes in craniofacial syndromes	Tokumaru AM et al. [8]	1996	In patients with Apert syndrome, temporal lobe deformities including adjacent temporal horn and atypical gyroscopic pattern bilaterally in temporal lobes, were identified.
5	Medial temporal lobe dysgenesis in Muenke syndrome and hypochondroplasia	Grosso et al. [14]	2003	Hypochondroplasia and Muenke syndrome due to FGFR3 mutations are connected with bilateral dysgenesis of the medial and temporal lobes.
6	Muenke syndrome with pigmentary disorder and probable hemimegalencephaly: an expansion of the phenotype	Abdel-Salam GM et al. [15]	2011	Individuals with Muenke syndrome due to mutations in FGFR3 gene presented left hemimegalencephaly.
7	A patient with Muenke syndrome manifesting migrating neonatal seizures	Okubo Y et al. [9]	2017	A typical symptom of Muenke syndrome is the deformed temporal lobes.
8	Internal brain herniation in a patient with Apert's syndrome	Wider TM et al. [16]	1995	In a case of syndromic craniosynostosis, a herniation of the frontal cortex was identified.
9	Monobloc and bipartition in craniofacial surgery	Cobb AR et al. [12]	2013	Treatment of syndromic craniosynostosis leads to frontal lobe changes.
10	Intracranial hypertension and cortical thickness in syndromic craniosynostosis	Wilson AT et al. [2]	2020	Syndromic craniosynostosis presents the correlation between intracranial hypertension and papilledema and thinner cortices.
11	Beare-Stevenson syndrome: two Dutch patients with cerebral abnormalities	Barge-Schaapveld DQ et al. [17]	2011	Beare-Stevenson syndrome is caused by FGFR2 gene mutations and it is characterized by simplified gyroscopic patterns.
12	Neurological functional connectivity in unilateral coronal synostosis: a side-based comparison	Park KE et al. [10]	2021	Non-syndromic unicoronal craniosynostosis leads to altered neural connectivity and the sidedness of the prematurely closed suture affects the consequences on the cortical connectivity.
13	Understanding the learning disabilities linked to sagittal craniosynostosis	Cabrejo R et al. [4]	2019	Sagittal non-syndromic craniosynostosis results in degenerated connectivity between cortical areas.
14	Altered brain connectivity in sagittal craniosynostosis	Beckett JS et al. [3]	2014	Occipital non-syndromic craniosynostosis leads to decreased neural function.
15	Structural brain differences in school-age children with and without single-suture craniosynostosis	Aldridge K et al. [11]	2017	Altered gray matter volume is identified as a result of occipital metopic non-syndromic craniosynostosis.
16	Quantitative analysis of cerebrospinal fluid spaces in children with occipital plagiocephaly	Sawin PD et al. [5]	1996	Non-syndromic occipital plagiocephaly can increase Sylvian fissure’s volume.

Fetuses born with Apert syndrome, a syndrome associated with the premature closure of the coronal suture due to mutations in the FGFR2 gene, display deformities in the temporal lobe. All examined fetuses showed hyperextension, oversulcation, and clefts in the temporal lobe region. Finally, temporal lobe abnormalities have also been described in cases of thanatophoric dysplasia, achondroplasia, and hypochondroplasia caused by mutations in FGFR3, as well as in Pfeiffer syndrome caused by a mutation in FGFR2 [6].

Furthermore, the authors report that after conducting a brain MRI scan in a fetus with Apert syndrome, at 23 weeks and two days gestational age, brachyturricephaly, overconvolution, and abnormal sulci in the lower and middle temporal lobe region were identified. In normally developed fetuses, the temporal sulci are visible on MRI examination after 26 weeks (superior temporal sulcus) and 32-33 weeks of gestation (inferior temporal sulcus). Therefore, the identification of overconvolution or clefts of the inferior temporal lobe on second-trimester MRI imaging is clearly an abnormality [7]. Another study reports that four patients with Apert syndrome had temporal lobe abnormalities, including the adjacent temporal horn, which were detected by thin-section coronal and occipital MRI performed in these patients [8].

In addition, in patients with hypochondroplasia and Muenke's syndrome with coronal craniosynostosis, due to a mutation in the FGFR3 gene, bilateral dysgenesis of the medial temporal lobes was observed. In particular, the MRI performed showed a defective gyroscopic pattern (cortical gyrus pattern) and abnormal temporal lobes. In these cases, an abnormal shape with thickening of the grey matter and incomplete differentiation between white and grey matter was observed. According to the authors, insufficient development of the medial temporal lobes could be related to abnormal skull formation [14].

At the initiative of other researchers, a two-year-old boy suffering from Muenke syndrome and craniosynostosis involving the right coronal, the sagittal, and the lamboid suture was examined. Molecular analysis revealed a mutation in the FGR3 gene, and an MRI scan performed revealed a left hemimegalencephaly. Hemimegalencephaly is a rare, sporadic malformation of the brain connected with the overdevelopment of one cerebral hemisphere. In addition, an MRI was performed at birth, and the axial view showed left hemimegalocephaly with asymmetry, dilatation, and elevation of the frontal horn and cortical dysplasia. The coronal view showed enlargement of the left cerebral hemisphere, and cortical dysplasia was also evident [15].

A different group of authors conducted three-dimensional (3D) computed tomography and repeated MRI scans on a one-year-old patient suffering from Muenke's syndrome. The boy presented with craniosynostosis with premature closure of the left coronal suture and deformed temporal lobes [9]. 

In another study, authors describe the case of a patient with syndromic craniosynostosis presenting with brachycephaly, diagnosed after a computed tomography scan performed preoperatively. They suggested that the patient's frontal cortex created a herniation within the bony prominence of the dura mater in the anterior cranial fossa [16].

Furthermore, it is reported that the frontofacial monobloc advancement method is used for the treatment of syndromic craniosynostosis. In 64% (32/50) of patients operated on using this technique, changes in the frontal cortex were observed. The majority of the changes observed were classified as "mild," with a change in frontal lobe volume of less than 5 cm³. It could be hypothesized that some frontal lobe changes are related to the "release" phenomenon caused by the rapid expansion of the frontal lobes, which were previously compressed due to the narrow anterior cavity. However, the precise anatomical site of the changes observed by the authors suggests that the changes are probably related to the technique used [12].

A study was conducted to evaluate the effect of risk factors for intracranial hypertension on cortical thickness in patients with syndromic craniosynostosis. As a subcomponent of volume, cortical thickness reflects the density of neurons per column, dendritic branches, and glial support. Cortical development occurs rapidly during childhood and adolescence. The model used showed a significant correlation between a history of papilledema and cortical thickness (p=0.036). Patients with hydrocephalus also appeared to have significantly thinner cortices (p=0.007). Furthermore, it has been shown that optic disc edema does not occur as a result of transient increases in intracranial pressure (ICH) but rather results from prolonged exposure to elevated ICH values. The necessity of this prolonged exposure to detect papilledema could, in contrast to other measurements, explain why edema occurs in the context of accelerated cortical thinning [2].

According to a study conducted in individuals with mutations in the FGFR gene, even though increased intracranial volume was observed, the cortical surface expansion in the parietal and occipital lobes was reduced. Specifically, patients with mutations in the FGFR gene showed an average of 201 cm³ greater intracranial volume (ICV) compared to subjects without craniosynostosis. However, these persons, despite the larger ICV, did not show a typical increase in cortical surface area. The cerebral cortex’s growth depends primarily on an increase in its surface, which scales disproportionately as brain size rises. Disorders that occur while the brain surface is growing have been associated with various pathologies that are involved with syndromic craniosynostosis, including cognitive impairment. Analysis of the lobes revealed that the correlation between ICV and the cerebral cortex was drastically reduced in the parietal and occipital lobes. Therefore, it is concluded that they have less surface space according to their volume [1].

In addition, it is beneficial to mention the case of two patients with Beare-Stevenson syndrome and anatomical brain malformations. This syndrome is caused by mutations in the fibroblast growth factor receptor gene (FGFR2). The patients underwent computed tomography, which indicated the closure of all sutures except a proximal part of the coronal suture, the frontal suture, and the peripheral part of the lambdoid sutures. Lastly, postmortem MRI revealed a simplified gyroscopic pattern [17].

A different patient with Apert syndrome presented an atypical gyroscopic pattern bilaterally in the temporal lobes, without any obvious neurocortical anomalies. As a result, the most severe gyroscopic anomalies were in patients with the most severe cranial malformations and, in particular, in those with Crouzon syndrome. Finally, although polymicrogyria is rarely mentioned in craniofacial anomalies, according to the authors, most cases of gyroscopic anomalies occur primarily as a result of cortical deformation due to cranial anomalies [8].

Non-syndromic Craniosynostosis

This case of the disease is related to the premature fusion of the cranial sutures in non-heritable cases [3,5].

These changes in the cerebral cortex may include various abnormalities in the connectivity between different areas of the cortex [10]. In particular, the study aimed to highlight the connectivity changes in the cortex of patients with non-syndromic unicoronal craniosynostosis, which was helpful in grasping the importance of the prematurely closed suture’s part in these abnormalities. The participants were 12 patients older than seven years old and an equal number of healthy controls. All of the patients presented reduced connectivity in areas related to visuomotor coordination and language function, belonging to the parietal and temporal cortex, as well as deficits in the prefrontal cortex that are normally associated with executive actions [10]. The scientists emphasized the differences between patients with right unicoronal craniosynostosis and healthy subjects. The former showed intrinsically altered neural connectivity in cortical parts responsible for complex mobility, proprioception, and motor skill development. In fact, the correlation between the early suture fusion sidedness and the consequences of the disease became clear. Patients with left unicoronal craniosynostosis were characterized by degenerated connectivity between the right primary visual cortex (Brodmann area 17) and the bilateral superior parietal lobe (Brodmann area 7), the two cunei, both primary visual cortexes (Brodmann area 17), and the two middle occipital gyri (Brodmann area 18). Altered connectivity was detected among the left middle frontal gyrus (Brodmann area 6) and the right superior parietal lobe (Brodmann area 7). In addition, deficient connections of the parietal and occipital areas related to movement coordination, and visual, and language function were revealed. More details about the alterations of the neural connectivity are listed in Table 4 [10].

**Table 4:** Altered cortical connectivity using intrinsic connectivity distribution analysis and seed-connectivity analysis Reported by Park KE et al. 2021 [10]

Methods	Population	Results
Intrinsic connectivity distribution analysis	All patients with non-syndromic unicoronal craniosynostosis were compared to controls	The analysis revealed weaker signal in the left superior parietal lobe (Brodmann area 7), bilateral angular gyri (Brodmann area 39), and right anterior transverse temporal area (Brodmann area 41).
Intrinsic connectivity distribution analysis	Patients with right non-syndromic unicoronal craniosynostosis were compared to controls	The analysis proved strong signal in the right cuneus, right primary visual cortex (Brodmann area 17), right parahippocampal gyrus (Brodmann area 36), right inferior temporal gyrus (Brodmann area 20), and right fusiform gyrus (Brodmann area 37) and weak signal in the right supramarginal gyrus (Brodmann area 40), the right anterior central gyrus (Brodmann area 6,) and the right superior parietal lobe (Brodmann area 7).
Intrinsic connectivity distribution analysis	Patients with left non-syndromic unicoronal craniosynostosis were compared to controls and patients with right non-syndromic unicoronal craniosynostosis	No modifications were found.
Seed-connectivity analysis	Patients with right non-syndromic unicoronal craniosynostosis were compared to controls	The analysis revealed impaired connectivity not only between the right cuneus (Brodmann area 18) and the right middle occipital groove (Brodmann area 19) but also among the anterior cingulate cortex and the right fusiform gyrus.
Seed-connectivity analysis	Patients with left non-syndromic unicoronal craniosynostosis were compared to controls and patients with right non-syndromic unicoronal craniosynostosis	The analysis showed abnormal neural connectivity between the bilateral cuneus, the right lingual gyrus (Brodmann area 19), and the left middle frontal gyrus (Brodmann area 6).

The data presented enriches the findings of previous studies. Among these, there are two examples that stand out. The first one used fMRI to identify reduced connectivity in the prefrontal cortex, middle temporal gyrus, and posterior cingulate gyrus of patients with non-syndromic unicoronal craniosynostosis, possibly leading to impaired emotional regulation and executive abilities in childhood. In the second one, intrinsic connectivity distribution analysis was applied to demonstrate strong signals in temporal lobe regions of unicoronal craniosynostosis patients, according to healthy subjects when both groups were subjected to emotional frustration [10].

Disorders in neural connectivity were also revealed in another study aimed at documenting neurocognitive impairment. The participants were adolescents with sagittal non-syndromic craniosynostosis who had undergone total skull vault cranioplasty. These patients were compared to teenagers dealing with attention deficit hyperactivity disorder (ADHD) sufferers and to healthy peers, the control group. Data from functional magnetic resonance imaging (fMRI) in combination with subsequent statistical analysis indicated reduced connectivity in patients with craniosynostosis compared to the control group, between Brodmann area 21 (BA21) in the left cerebral hemisphere (associated with language and auditory processing), Brodmann area 19 (BA19, related to visual stimulus processing), and the visual association cortex [4].

Even more specific information about the neural connections in the cortex was obtained in a study that focused on patients 12-13 years old with occipital non-syndromic craniosynostosis (treated by cranioplasty vault surgery) and healthy peers. The scientists concentrated on highlighting the influence of functional and structural connectivity variations on sufferers’ neurocognitive behavior. Many analyses (e.g., fMRI) revealed such variations in several regions of the cerebral cortex. In particular, reduced neural interaction was revealed between Brodmann area 8, the prefrontal cortex, and the frontal part of the cingulate cortex in the patients. The connectivity was also incomplete between Brodmann areas 7, 39, and 40; the posterior part of the cingulate cortex; the prefrontal lobe; the supramarginal, left angular, lingual, and fusiform gyri. Similar modifications were found among the connections of the left and right cingulate cortex and the right hemisphere prefrontal lobe, while altered connectivity between the left ventromedial prefrontal cortex and the right middle frontal gyrus was also detected. This data confirms that the abnormal cortical neural interactions are associated with the neurocognitive disorders of the patient participants. As a result, areas involved in visual and spatial processing (prefrontal lobe, posterior cingulate cortex) and language processing (left supramarginal, left angular, lingual, and fusiform gyrus) are affected [3].

Furthermore, it is important to investigate another study that focuses on the quantitative brain characteristics’ potential changes in children with both occipital and metopic non-syndromic craniosynostosis (surgically treated) compared to healthy controls [11]. In fact, differences in the total and the individual gray matter volume between these two groups of patients were investigated, such as whether the type of the prematurely fused suture (sagittal or metopic) determines those alterations. Deviation among the affected persons and the controls, taking into consideration the volumes of the whole brain (WBV), the cortical gray matter (CGV), the frontal cortical gray matter (FGV), the temporal cortical gray matter (TGV), and the parietal-occipital cortical gray matter (POGV), was proved by using functional MRI. However, this deviation was not statistically significant (p-value > 0.05), although it is worth mentioning it for a deeper understanding of the phenomenon [11].

The increase of the Sylvian fissure’s volume is also mentioned as a cerebral cortical change caused by non-syndromic craniosynostosis. An important study that compared newborns who suffered from occipital plagiocephaly (premature lambdoid suture fusion) to healthy infants led to the revelation that the volume of the patients’ Sylvian fissures was significantly higher than the healthy infants’ fissures’ volume. More specifically, a difference of 5.1 mL was identified while the patients’ volume and the controls’ were 5.8 mL and 0.7 mL, respectively (p-value<0.0001). Finally, the deviation between the volume of the patient’s lateral fissures was recorded, as the volume of the unilateral fissure with the prematurely fused suture appeared to have a smaller size (4.5 mL versus 7.1 mL of the contralateral fissure with p-value=0.001) [5].

## Conclusions

Craniosynostosis is associated with changes in the brain cortex’s thickness, surface area, connectivity between different regions of the brain, and the morphology of fissures and gyri. Two theories describe this association. According to the first one, called the deformation theory, cranial dysmorphology as a consequence of craniosynostosis causes abnormalities in the brain at a secondary level. The second theory called the malformation theory, argues that craniosynostosis occurs in direct association with primary neural alterations. Existing data are not sufficient to resolve the dilemma, as each theory is verified when examining different brain regions. In this context, it would also be useful to report potential treatments. The therapeutic approach differs depending on the type of craniosynostosis, and while surgery remains the most common treatment option, groundbreaking rehabilitation modalities are currently rising. These include applications of regenerative medicine with mesenchymal stem cell transplantation and pharmacological treatments.

## Appendices

Appendix 1 

**Table 5:** PRISMA checklist PRISMA: Preferred Reporting Items for Systematic Reviews and Meta-Analyses

Section and topic	Item #	Checklist item	Location where the item is reported
TITLE			
Title	1	Identify the report as a systematic review.	
ABSTRACT			
Abstract	2	See the PRISMA 2020 for the Abstract checklist.	Abstract
INTRODUCTION			
Rationale	3	Describe the rationale for the review in the context of existing knowledge.	3
Objectives	4	Provide an explicit statement of the objective(s) or question(s) the review addresses.	3
METHODS			
Eligibility criteria	5	Specify the inclusion and exclusion criteria for the review and how studies were grouped for the syntheses.	5,6
Information sources	6	Specify all databases, registers, websites, organizations, reference lists, and other sources searched or consulted to identify studies. Specify the date when each source was last searched or consulted.	5
Search strategy	7	Present the full search strategies for all databases, registers, and websites, including any filters and limits used.	4,5,6
Selection process	8	Specify the methods used to decide whether a study met the inclusion criteria of the review, including how many reviewers screened each record and each report retrieved, whether they worked independently, and if applicable, details of automation tools used in the process.	4,5
Data collection process	9	Specify the methods used to collect data from reports, including how many reviewers collected data from each report, whether they worked independently, any processes for obtaining or confirming data from study investigators, and if applicable, details of automation tools used in the process.	4,5,6
Data items	10a	List and define all outcomes for which data were sought. Specify whether all results that were compatible with each outcome domain in each study were sought (e.g. for all measures, time points, analyses), and if not, the methods used to decide which results to collect.	4
	10b	List and define all other variables for which data were sought (e.g. participant and intervention characteristics, funding sources). Describe any assumptions made about any missing or unclear information.	4,5,6
Study risk of bias assessment	11	Specify the methods used to assess the risk of bias in the included studies, including details of the tool(s) used, how many reviewers assessed each study, and whether they worked independently, and if applicable, details of automation tools used in the process.	4,5
Effect measures	12	Specify for each outcome the effect measure(s) (e.g. risk ratio, mean difference) used in the synthesis or presentation of results.	5
Synthesis methods	13a	Describe the processes used to decide which studies were eligible for each synthesis (e.g. tabulating the study intervention characteristics and comparing against the planned groups for each synthesis (item #5)).	5,6
	13b	Describe any methods required to prepare the data for presentation or synthesis, such as handling of missing summary statistics, or data conversions.	5,6
	13c	Describe any methods used to tabulate or visually display the results of individual studies and syntheses.	5,6
	13d	Describe any methods used to synthesize results and provide a rationale for the choice(s). If meta-analysis was performed, describe the model(s), method(s) to identify the presence and extent of statistical heterogeneity, and software package(s) used.	5,6
	13e	Describe any methods used to explore possible causes of heterogeneity among study results (e.g. subgroup analysis, meta-regression).	5,6
	13f	Describe any sensitivity analyses conducted to assess the robustness of the synthesized results.	5,6
Reporting bias assessment	14	Describe any methods used to assess the risk of bias due to missing results in a synthesis (arising from reporting biases).	4,5,6
Certainty assessment	15	Describe any methods used to assess certainty (or confidence) in the body of evidence for an outcome.	5,6
RESULTS			
Study selection	16a	Describe the results of the search and selection process, from the number of records identified in the search to the number of studies included in the review, ideally using a flow diagram.	7
	16b	Cite studies that might appear to meet the inclusion criteria, but which were excluded, and explain why they were excluded.	Flowchart (but it is in the methods section)
Study characteristics	17	Cite each included study and present its characteristics.	Table 2 (but it is in the discussion section)
Risk of bias in studies	18	Present assessments of risk of bias for each included study.	7
Results of individual studies	19	For all outcomes, present, for each study: (a) summary statistics for each group (where appropriate) and (b) an effect estimate and it's precision (e.g. confidence/credible interval), ideally using structured tables or plots.	Table 1
Results of syntheses	20a	For each synthesis, briefly summarise the characteristics and risk of bias among contributing studies.	7
	20b	Present results of all statistical syntheses conducted. If meta-analysis was done, present for each the summary estimate and its precision (e.g. confidence/credible interval) and measures of statistical heterogeneity. If comparing groups, describe the direction of the effect.	7
	20c	Present results of all investigations of possible causes of heterogeneity among study results.	7
	20d	Present results of all sensitivity analyses conducted to assess the robustness of the synthesized results.	7
Reporting biases	21	Present assessments of risk of bias due to missing results (arising from reporting biases) for each synthesis assessed.	7
Certainty of evidence	22	Present assessments of certainty (or confidence) in the body of evidence for each outcome assessed.	7
DISCUSSION			
Discussion	23a	Provide a general interpretation of the results in the context of other evidence.	Table 2
	23b	Discuss any limitations of the evidence included in the review.	Table 4
	23c	Discuss any limitations of the review processes used.	Table 4
	23d	Discuss the implications of the results for practice, policy, and future research.	27
OTHER INFORMATION			
Registration and protocol	24a	Provide registration information for the review, including the register name and registration number, or state that the review was not registered.	-
	24b	Indicate where the review protocol can be accessed, or state that a protocol was not prepared.	-
	24c	Describe and explain any amendments to information provided at registration or in the protocol.	-
Support	25	Describe sources of financial or non-financial support for the review, and the role of the funders or sponsors in the review.	-
Competing interests	26	Declare any competing interests of review authors.	-
Availability of data, code, and other materials	27	Report which of the following are publicly available and where they can be found: template data collection forms; data extracted from included studies; data used for all analyses; analytic code; any other materials used in the review.	-

## References

[1] Wilson AT, Den Ottelander BK, Van Veelen MC (2022). Cerebral cortex maldevelopment in syndromic craniosynostosis. Dev Med Child Neurol.

[2] Wilson AT, Den Ottelander BK, De Goederen R (2020). Intracranial hypertension and cortical thickness in syndromic craniosynostosis. Dev Med Child Neurol.

[3] Beckett JS, Brooks ED, Lacadie C (2014). Altered brain connectivity in sagittal craniosynostosis. J Neurosurg Pediatr.

[4] Cabrejo R, Lacadie C, Brooks E (2019). Understanding the learning disabilities linked to sagittal craniosynostosis. J Craniofac Surg.

[5] Sawin PD, Muhonen MG, Menezes AH (1996). Quantitative analysis of cerebrospinal fluid spaces in children with occipital plagiocephaly. J Neurosurg.

[6] Stark Z, McGillivray G, Sampson A (2015). Apert syndrome: temporal lobe abnormalities on fetal brain imaging. Prenat Diagn.

[7] Quintas-Neves M, Soares-Fernandes JP (2018). Fetal brain MRI in Apert syndrome: early in vivo detection of temporal lobe malformation. Childs Nerv Syst.

[8] Tokumaru AM, Barkovich AJ, Ciricillo SF, Edwards MS (1996). Skull base and calvarial deformities: association with intracranial changes in craniofacial syndromes. AJNR Am J Neuroradiol.

[9] Okubo Y, Kitamura T, Anzai M (2017). A patient with Muenke syndrome manifesting migrating neonatal seizures. Brain Dev.

[10] Park KE, Singh A, Lacadie C, Allam O, Smetona J, Alperovich M, Persing JA (2021). Neurological functional connectivity in unilateral coronal synostosis: a side-based comparison. J Craniofac Surg.

[11] Aldridge K, Collett BR, Wallace ER (2017). Structural brain differences in school-age children with and without single-suture craniosynostosis. J Neurosurg Pediatr.

[12] Cobb AR, Boavida P, Docherty R, Dunaway D, Saunders DE, Jeelani O, Hayward RD (2013). Monobloc and bipartition in craniofacial surgery. J Craniofac Surg.

[13] Henry BM, Tomaszewski KA, Ramakrishnan PK (2017). Development of the anatomical quality assessment (AQUA) tool for the quality assessment of anatomical studies included in meta-analyses and systematic reviews. Clin Anat.

[14] Grosso S, Farnetani MA, Berardi R (2003). Medial temporal lobe dysgenesis in Muenke syndrome and hypochondroplasia. Am J Med Genet A.

[15] Abdel-Salam GM, Flores-Sarnat L, El-Ruby MO (2011). Muenke syndrome with pigmentary disorder and probable hemimegalencephaly: an expansion of the phenotype. Am J Med Genet A.

[16] Wider TM, Schwartz TH, Carmel PW, Wood-Smith D (1995). Internal brain herniation in a patient with Apert's syndrome. Ann Plast Surg.

[17] Barge-Schaapveld DQ, Brooks AS, Lequin MH, van Spaendonk R, Vermeulen RJ, Cobben JM (2011). Beare-Stevenson syndrome: two Dutch patients with cerebral abnormalities. Pediatr Neurol.

